# Agreement and Systematic Differences Between Intraoperative ROSA Hip-Knee-Ankle Measurements and Standing Radiographic Alignment After Robotic-Assisted Total Knee Arthroplasty: A Retrospective Agreement Study

**DOI:** 10.3390/jcm15197432

**Published:** 2026-09-24

**Authors:** Wei-Cheng Tseng, Ching-Heng Lin, Yu-Tsung Lin, Cheng-Chi Wang, Kao-Chang Tu, Kun-Hui Chen, Cheng-Hung Lee, Cheng-Jung Ho, Chung-Yuh Tzeng

**Affiliations:** 1 Department of Orthopedics, Kaohsiung Medical University Hospital, Kaohsiung Medical University, Kaohsiung City 807, Taiwan; wayne03010301@gmail.com (W.-C.T.); cjho@kmu.edu.tw (C.-J.H.); 2 Department of Medical Research, Taichung Veterans General Hospital, Taichung 407, Taiwan; epid@vghtc.gov.tw; 3 Department of Public Health, College of Medicine, Fu Jen Catholic University, New Taipei City 242, Taiwan; 4 Department of Industrial Engineering and Enterprise Information, Tunghai University, Taichung 407, Taiwan; 5 Institute of Public Health and Community Medicine Research Center, National Yang Ming Chiao Tung University, Taipei 112, Taiwan; 6 Department of Orthopedics, Taichung Veterans General Hospital, Taichung 407, Taiwan; dan90008@gmail.com (Y.-T.L.); ccwang827@gmail.com (C.-C.W.); shark310751@gmail.com (K.-C.T.); orthochen@gmail.com (K.-H.C.); leechenghung0115@gmail.com (C.-H.L.); 7 Department of Public Health, China Medical University, Taichung 406, Taiwan; 8 Department of Biomedical Engineering, Hung Kuang University, Taichung 433, Taiwan; 9 Department of Computer Science and Information Engineering, Providence University, Taichung 402, Taiwan; 10 Department of Post-Baccalaureate Medicine, College of Medicine, National Chung Hsing University, Taichung 433, Taiwan; 11 Department of Food Science and Technology, Hung Kuang University, Taichung 433, Taiwan; 12 Department of Nursing, Jenteh Junior College of Medicine, Nursing and Management, Miaoli 356, Taiwan

**Keywords:** robotic-assisted total knee arthroplasty, ROSA Knee System, hip-knee-ankle angle, agreement, Bland–Altman, component sizing, insert thickness

## Abstract

**Background/Objectives:** Intraoperative robotic hip-knee-ankle angle (HKA) measurements are obtained with the patient supine under operative conditions and may not directly represent postoperative standing weight-bearing alignment. This study quantified the correspondence and systematic differences between intraoperative ROSA HKA and standing radiographic HKA after robotic-assisted total knee arthroplasty. **Methods:** This retrospective single-center study included 208 knees from 178 consecutive patients, including 30 bilateral patients. The primary comparison was post-trial-component intraoperative ROSA HKA versus postoperative standing radiographic HKA; the secondary comparison was pre-cut intraoperative ROSA HKA versus preoperative standing radiographic HKA. Patient-cluster bootstrap confidence intervals used 10,000 resamples. Agreement was evaluated using Bland–Altman analysis, absolute-agreement ICC(A,1), and weighted kappa; Spearman ρ described association. **Results:** For the primary comparison, mean bias (ROSA minus radiographic HKA) was −1.75° (95% CI −2.08 to −1.43°), conventional limits of agreement were −6.36° to 2.85°, and ICC(A,1) was 0.472 (95% CI 0.379 to 0.557). Differences were within 3° in 70.2% of knees and exceeded 3° in 29.8%; proportional bias was present (slope −0.569, *p* < 0.001). The secondary comparison showed bias 0.97° and ICC(A,1) 0.956. Exact femoral and tibial size agreement was 42.3% and 80.8%, respectively. The standardized surgeon-entered 10 mm starting value was retained in 57.7% of knees. These systematic and angle-dependent differences may partly reflect the differing loading, positioning, and measurement conditions between the intraoperative trial construct and postoperative standing radiography. **Conclusions:** Post-trial-component ROSA HKA showed systematic and angle-dependent differences from postoperative standing HKA and should not be considered interchangeable with, or a fixed patient-level predictor of, postoperative standing alignment.

## 1. Introduction

Limb alignment is an important consideration in total knee arthroplasty (TKA), although the optimal postoperative alignment target remains debated. Native lower-limb alignment varies substantially among individuals, and pre-arthritic alignment may not be reliably reconstructed in knees with established osteoarthritis. Consequently, contemporary alignment philosophies differ in the relative emphasis placed on mechanical alignment, native anatomy, joint-line orientation, and soft-tissue balance [1,2]. Substantial malalignment has been associated with polyethylene wear, instability, revision, and inferior clinical outcomes [1,2,3,4,5]. Conventional TKA remains dependent on surgeon-controlled bone preparation and soft-tissue balancing. Variability in bone preparation and component positioning may contribute to component-positioning outliers, whereas variability in soft-tissue balancing may contribute to gap asymmetry, instability, abnormal knee kinematics, pain, and poorer patient-reported outcomes [3,6,7,8,9,10]. Intraoperative soft-tissue assessment and insert selection may also vary among surgeons; a cadaveric study reported a 1- to 2 mm variation in insert-height selection even among experienced arthroplasty surgeons [11]. Computer-assisted navigation and robotic-assisted TKA (RATKA) were developed to improve the reproducibility of bone preparation and component positioning while providing quantitative intraoperative assessment of alignment and soft-tissue balance [3,4,10,11,12,13,14,15].

The ROSA Knee System was used in its semiactive, image-based workflow, which supports preoperative planning, simulation of resection and alignment parameters, and real-time intraoperative gap assessment [1,2,4,5,10,16]. However, intraoperative and postoperative hip-knee-ankle angle (HKA) measurements are obtained under different biomechanical conditions. In the present workflow, post-trial-component intraoperative ROSA HKA was recorded with the patient supine, the knee in full extension, and the ankle positioned clinically at approximately 90°, without intentional axial loading or varus-valgus stress. Postoperative radiographic HKA was measured during standing weight bearing after final implantation. An association between these measurements therefore does not establish agreement, and intraoperative ROSA HKA should not be assumed to reproduce postoperative standing alignment. Previous robotic TKA studies have focused primarily on bone-cut execution, component positioning, and alignment outliers, whereas evidence regarding cross-context agreement between intraoperative robotic HKA and postoperative standing HKA remains limited and has largely involved other robotic platforms or imaging conditions [17,18]. Quantifying the difference between these measurements may help surgeons understand how closely intraoperative ROSA HKA reflects postoperative standing HKA and avoid treating the two measurements as interchangeable.

Robotic workflows also generate preoperative component-size plans and permit an initial insert-thickness setting, both of which may be revised according to intraoperative anatomy, gap balance, and stability. Planned-versus-implanted component-size agreement therefore describes concordance between preoperative planning and final intraoperative selection, whereas adjustment from the standardized surgeon-entered 10 mm starting value reflects individualized intraoperative balancing rather than system prediction error. The primary objective of this study was to evaluate agreement and systematic differences between post-trial-component intraoperative ROSA HKA and postoperative standing radiographic HKA. Secondary objectives were to assess agreement between pre-cut intraoperative ROSA HKA and preoperative standing radiographic HKA, concordance between planned and implanted femoral and tibial component sizes, and adjustment from the standardized surgeon-entered 10 mm starting value. We hypothesized that intraoperative ROSA HKA and postoperative standing radiographic HKA would be associated but not directly interchangeable because they are obtained under different measurement conditions.

## 2. Materials and Methods

### 2.1. Study Design and Participants

This retrospective observational single-center study included all consecutive ROSA-assisted primary total knee arthroplasties performed for primary knee osteoarthritis from 8 July 2022 to 29 July 2025. All consecutive cases meeting this study definition during the predefined period were included, and no additional exclusions were applied. A total of 178 patients comprising 208 knees were included in the final analysis, including 30 patients who underwent simultaneous bilateral total knee arthroplasty (Figure 1). Knee-specific outcomes were analyzed at the knee level, with within-patient dependence accounted for in all inferential analyses. No a priori sample-size calculation was performed. The sample size was fixed by the number of consecutive cases available during the predefined retrospective study period. Statistical precision was characterized using patient-cluster bootstrap 95% confidence intervals for the mean bias, limits of agreement, and other agreement estimates. The study was conducted in accordance with the Declaration of Helsinki and approved by the Institutional Review Board of Taichung Veterans General Hospital (protocol CE24151B; Date of Approval: 17 April 2024, protocol CE24151B-1; Date of Approval: 5 March 2025, protocol CE24151B-2; Date of Approval: 12 February 2026). The Institutional Review Board waived informed consent because of the retrospective use of clinical data.

### 2.2. Surgical Procedure and Implants

All procedures were performed by a single surgeon with more than 20 years of experience in conventional total knee arthroplasty and approximately 4 years of experience with ROSA-assisted total knee arthroplasty through a medial parapatellar approach using the ROSA Knee System (Zimmer Biomet, Warsaw, IN, USA) and the Persona^®^ The Personalized Knee^®^ System (Zimmer Biomet, Warsaw, IN, USA). ROSA was used in its image-based workflow based on standing full-length radiographs and intraoperative registration. Planned femoral and tibial component sizes for the planned-versus-implanted agreement analyses were recorded after completion of the preoperative image-based plan and before surgery. After tracker placement and instrument calibration, the femoral head center was identified from multiple static positions of the hip and, together with the femoral axis point at the deepest part of the intercondylar notch, was used to establish the femoral mechanical axis. The distal femoral condyles were registered to determine distal resection depth; the posterior condylar, transepicondylar, and anteroposterior axes served as femoral rotational references; and the anterior femoral cortex was registered for femoral sizing and assessment of potential anterior notching. The tibial mechanical axis was established using the tibial axis point at the intramedullary canal entrance and the ankle center derived from the medial and lateral malleolar landmarks. The medial and lateral tibial plateau points determined the proximal tibial resection level, while the PCL insertion and medial third of the tibial tubercle provided a tibial rotational reference.

A functional alignment strategy with predefined component-level coronal boundaries relative to the respective femoral and tibial mechanical axes was used. The initial coronal plan placed the distal femoral and proximal tibial resections perpendicular to their respective mechanical axes. Following completion of bony registration, pre-cut HKA was recorded before meniscal removal, big osteophyte removal, or bone resection, without intentional axial loading or varus-valgus stress. The medial and lateral menisci and accessible osteophytes were subsequently removed, after which medial and lateral gap behavior was assessed under manually applied varus-valgus stress at 0° of extension and 90° of flexion. The initial ROSA plan was modified when necessary according to the intraoperative gap pattern rather than a predefined preoperative HKA threshold. In varus knees with persistent medial tightness, the tibial resection could be adjusted up to 2° of varus relative to the tibial mechanical axis, with approximately 1–2° of femoral coronal adjustment in either direction. In valgus knees with persistent lateral tightness or gap asymmetry, the distal femoral position could be adjusted up to 3° of valgus relative to the femoral mechanical axis, with up to 2° of tibial varus when required. Femoral rotation was initially based on the ROSA image-based plan and subsequently refined according to the flexion-gap pattern. Tibial slope was individualized according to implant design and flexion-extension balance rather than assigned a uniform target. In this surgeon’s workflow, a medial-to-lateral gap difference of approximately 2 mm or less was generally accepted [16,19], and complete compartmental symmetry was not required [20]. These independent coronal adjustments changed the planned orientations of the respective femoral and tibial components and were therefore expected to influence post-trial HKA. However, because quantitative post-resection angular validation was not performed, the achieved cut-plane orientations could not be confirmed. The measured post-trial HKA also reflected the trial construct, passive soft-tissue constraints, and limb positioning under supine, non-weight-bearing conditions.

Soft-tissue release was minimized and used selectively when satisfactory balance could not be achieved through component-position or bone-resection adjustments. An overall HKA within ±3° of neutral was preferred but was not an absolute intraoperative constraint; residual alignment outside this range was accepted when satisfactory flexion-extension balance and knee stability were achieved. The robotic arm positioned the cutting guide, after which bone cuts were performed using a conventional oscillating saw. Resected bone thickness was checked using a Vernier caliper. The ROSA post-resection cut-validation workflow was not performed for either the distal femoral or proximal tibial resection; therefore, achieved coronal and sagittal cut-plane angles were not quantitatively recorded. After trial implantation, balance was reassessed using ROSA gap information together with the surgeon’s clinical assessment. No separate tibial pressure-sensor device was used. Posterior-stabilized (PS) or medial-congruent (MC) inserts were paired with the corresponding femoral component design; 140 knees received PS constructs and 68 received MC constructs. Construct selection was based primarily on patient preference after discussion of implant options and associated out-of-pocket costs, rather than on predefined anatomical or gap-balancing criteria. For every knee, the operating surgeon entered 10 mm as a standardized starting value in the ROSA planning display. This was neither a mandatory system default nor a system-generated patient-specific prediction. Final polyethylene insert thickness was determined intraoperatively after assessment of medial and lateral gaps, soft-tissue balance, and stability in both full extension and 90° of flexion and was permitted to differ from the initial 10 mm value.

### 2.3. Intraoperative ROSA HKA Measurements

Pre-cut intraoperative ROSA HKA was recorded after completion of bony registration but before meniscal removal, osteophyte removal, or bone resection. The patient was supine, and the operating surgeon positioned the knee in full extension. No intentional axial loading or varus-valgus stress was applied during HKA acquisition; the patella remained in its native position, and the tourniquet was inflated. A single measurement was recorded.

Post-trial-component intraoperative ROSA HKA was recorded after completion of final soft-tissue balancing during the final trial reduction, with trial femoral and tibial components and a trial polyethylene insert in place. The trial insert thickness was identical to the final implanted insert thickness. The patient remained supine, with the knee in full extension and the ankle positioned clinically at approximately 90°. No intentional axial loading or varus-valgus stress was applied during HKA acquisition; the patella was reduced, and the tourniquet remained inflated. A rigid positioning device or goniometer was not used to standardize or verify ankle position. A single measurement was recorded at the same operative time point in every knee. After this measurement, no further changes were made to component position or insert thickness. The value therefore represented the final balanced trial construct rather than a direct measurement of the cemented final construct. The HKA agreement analyses evaluated coronal alignment only in full extension.

### 2.4. Radiographic Acquisition and HKA Measurement

Conventional standing full-length lower-extremity radiographs were acquired with patients instructed to distribute weight equally between both limbs, with the patellae directed forward and the knees in maximal extension. Limb rotation and foot position were adjusted to achieve appropriate frontal-plane positioning. Radiographs were considered suitable for HKA measurement when both limbs were weight bearing, the knees were in maximal extension, the patellae were directed forward, and no evident limb rotation or asymmetric weight bearing was present. Images not meeting these positioning requirements were repeated before measurement. A formal numerical rotation tolerance, objective measure of limb loading, and source-to-detector distance were not available as study variables in this retrospective dataset. Postoperative radiographs were obtained approximately one month after surgery at the second postoperative outpatient visit. HKA was defined as the angle between the femoral mechanical axis, extending from the femoral head center to the knee center, and the tibial mechanical axis, extending from the knee center to the ankle center. The centers of the hip, knee, and ankle were marked manually. Positive HKA denoted varus and negative HKA denoted valgus. Standing full-length hip-to-ankle radiography has been shown to provide reliable and reproducible coronal lower-limb alignment measurements, with high repeatability and intraobserver/interobserver agreement reported for mechanical-axis measurements [21,22,23,24]. Radiographic HKA measurements were performed by a third-year orthopedic resident with approximately 5 years of orthopedic research experience. The observer was an author of the study. Based on the operative team’s recollection, the observer had participated as a surgical assistant during approximately the first 15–20 ROSA-assisted TKA procedures but did not participate in subsequent ROSA procedures after leaving the institution. Because case-level participation records were unavailable in this retrospective dataset, the exact number and identity of these knees could not be reliably reconstructed. Before measurement, patient names, medical record numbers, examination dates, and other identifying information were removed from the radiographs, which were relabeled using nonidentifying study codes. The observer did not have access to the code linkage, clinical information, or corresponding ROSA values during measurement. These safeguards reduced the possibility of case recognition but could not ensure complete blinding. Landmark placement and the resulting HKA measurement were subsequently reviewed for quality control by an orthopedic surgeon; this review was not a prespecified independent repeated measurement. Both observers had received the same HKA-measurement training. Radiographs were acquired using a Siemens Ysio Max system (Siemens Healthineers, Erlangen, Germany), and HKA measurements were performed using Smart Iris V.1.3.6.0 PACS/measurement software (Smart Iris, Taiwan).

### 2.5. Outcomes

The primary outcome was agreement between post-trial-component intraoperative ROSA HKA and postoperative standing radiographic HKA. The secondary HKA outcome was agreement between pre-cut intraoperative ROSA HKA and preoperative standing radiographic HKA. Additional outcomes were femoral and tibial component-size agreement and adjustment of implanted insert thickness from the standardized surgeon-entered 10 mm starting value. For exploratory category analyses, HKA was classified using a commonly used mechanical-alignment threshold as valgus (<−3°), neutral (−3° to +3°), or varus (>+3°). No clinically acceptable HKA-difference or limits-of-agreement threshold was prespecified.

### 2.6. Statistical Analysis

Continuous baseline and HKA variables were summarized using mean ± standard deviation, whereas medians and interquartile ranges were additionally reported for selected absolute-difference measures. Categorical variables were summarized as counts and percentages. For the Bland–Altman analyses, the distributions of paired differences were assessed using histograms and normal Q-Q plots, supplemented by skewness, excess kurtosis, and the Shapiro–Wilk test. Because formal normality tests may be sensitive to minor distributional departures in samples of this size, graphical distribution and tail behavior were considered together with the formal test rather than relying on the *p*-value alone. HKA differences were defined as ROSA minus radiographic HKA. Conventional Bland–Altman mean bias and 95% limits of agreement (LoA) were calculated as the mean difference ± 1.96 standard deviations. As a distributional sensitivity analysis, empirical 2.5th and 97.5th percentiles of the paired differences were also examined. Patient-cluster bootstrap resampling with 10,000 resamples was used to obtain 95% confidence intervals while accounting for within-patient dependence in bilateral procedures. Absolute-agreement ICC(A,1), corresponding to ICC(2,1), quantified absolute agreement; Spearman ρ was reported as an association measure. Mean and median absolute differences and proportions within 1°, 2°, and 3° were calculated. Proportional bias was evaluated by regressing the difference on the paired mean using patient-cluster-robust standard errors. Regression-based expected differences at selected paired-mean values were treated as exploratory and reported in the Supplementary Materials; conventional LoA remained the primary agreement summary. Component-size analyses included exact agreement, directional disagreement, mean absolute size-level difference, agreement within one and two size levels, linearly weighted kappa, and Spearman ρ. Bowker symmetry was reserved for the Supplementary Materials and assessed using 10,000 patient-cluster permutations. Insert adjustment was analyzed exploratorily with patient-cluster-robust linear and logistic models. A fixed-seed one-knee-per-patient sensitivity analysis was supplemented by 1000 repeated random one-knee selections. There were no missing values for the variables included in the primary and secondary analyses; therefore, no imputation was performed. No multiplicity correction was applied to exploratory analyses. Two-sided *p* < 0.05 was considered statistically significant. Analyses were performed in Python 3.12.13. The analysis code and aggregate outputs were independently reviewed by Wei-Cheng Tseng and Ching-Heng Lin.

### 2.7. Data Governance

Analyses were conducted using pseudonymized study data. The patient linkage field was used only to account for bilateral procedures, and direct identifiers were excluded from analytic and reporting outputs. OpenAI Codex (OpenAI 5.6, San Francisco, CA, USA; accessed in 2026) was used to assist with analysis-code development, code checking, aggregate table and figure generation, and language revision. All analyses, numerical outputs, and interpretations were reviewed and validated by the authors.

## 3. Results

### 3.1. Participants and Baseline Characteristics

All 178 consecutive patients (208 knees) were analyzed, including 30 patients who underwent simultaneous bilateral total knee arthroplasty. Patient- and knee-level characteristics are summarized in Table 1. The cohort predominantly demonstrated preoperative varus alignment.

### 3.2. Post-Trial-Component HKA Agreement and Sensitivity Analysis

Post-trial-component intraoperative ROSA HKA averaged 1.03 ± 1.90°, whereas postoperative standing radiographic HKA averaged 2.79 ± 3.09°. Mean bias was −1.75° (95% CI −2.08 to −1.43°), with conventional LoA from −6.36° to 2.85°. ICC(A,1) was 0.472 (95% CI 0.379 to 0.557), and Spearman ρ was 0.626 (95% CI 0.520 to 0.715). Mean absolute difference was 2.23°; absolute differences were within 1°, 2°, and 3° in 34.1%, 54.3%, and 70.2% of knees, respectively (Table 2). Although individual differences occurred in both directions, they were not centered on zero. Absolute differences exceeded 2° in 45.7% and 3° in 29.8% of knees. The Bland–Altman plot with the proportional-bias regression is shown in Figure 2. In the exploratory ±3° categorical analysis, post-trial ROSA and postoperative standing radiographic HKA were classified in the same valgus/neutral/varus category in 121 of 208 knees (58.2%), whereas 87 knees (41.8%) changed category between measurement conditions. The most frequent discordant pattern was neutral alignment by ROSA followed by varus alignment on postoperative standing radiography (74 knees). The exploratory weighted kappa was 0.263. The complete confusion matrix is presented in Supplementary Table S2.

Proportional bias was present: the cluster-robust regression intercept was −0.667 (95% CI −1.004 to −0.330; *p* < 0.001), and the slope was −0.569 (95% CI −0.683 to −0.456; *p* < 0.001). Thus, the expected ROSA-minus-radiographic difference became more negative as the paired mean increased. Exploratory regression-based expected differences are reported in the Supplementary Materials. Selecting one knee per patient produced results consistent with the full clustered analysis; detailed fixed and repeated-selection estimates are provided in the Supplementary Materials. Distributional assessment showed only modest departures from normality in the paired differences. The empirical percentile limits (−6.68° to +2.64°) were similar to the conventional Bland–Altman limits (−6.36° to +2.85°), indicating that the distributional departure did not materially alter the interpretation of the primary agreement analysis (Supplementary Table S9).

### 3.3. Pre-Cut HKA Agreement

Pre-cut intraoperative ROSA HKA averaged 8.16 ± 6.81°, whereas preoperative standing radiographic HKA averaged 7.19 ± 6.61°. Mean bias was 0.97° (95% CI 0.72 to 1.22°), with conventional LoA from −2.45° to 4.38°. ICC(A,1) was 0.956 (95% CI 0.937 to 0.969), and Spearman ρ was 0.954 (95% CI 0.931 to 0.968). The proportional-bias slope was 0.031 (95% CI −0.009 to 0.071; *p* = 0.124). Figure 3 presents the conventional Bland–Altman plot. For the secondary comparison, pre-cut ROSA and preoperative standing radiographic HKA were classified in the same category in 194 of 208 knees (93.3%), whereas 14 knees (6.7%) changed category. The exploratory weighted kappa was 0.855. The complete confusion matrix is presented in Supplementary Table S2. A concise summary of the primary and secondary HKA agreement estimates is presented in Table 2. Similarly, the empirical percentile limits for the secondary comparison (−2.61° to +4.57°) were close to the conventional Bland–Altman limits (−2.45° to +4.38°), supporting the robustness of the overall agreement interpretation (Supplementary Table S9).

### 3.4. Component-Size Agreement

Femoral component-size results are summarized in Table 3. Exact agreement was 42.3%; the planned size was smaller than the implanted size in 15.9% and larger in 41.8%. The mean absolute size-level difference was 0.69, 88.5% were within one level, and all knees were within two levels. Linearly weighted kappa was 0.631, whereas Spearman ρ was 0.851. The directional distribution indicates that planned femoral sizes were more often larger than smaller relative to the implanted size. Tibial component-size results are summarized in Table 4. Exact agreement was 80.8%; the planned size was smaller than the implanted size in 6.7% and larger in 12.5%. The mean absolute size-level difference was 0.21, linearly weighted kappa was 0.815, and Spearman ρ was 0.908.

### 3.5. Insert-Thickness Adjustment

The implanted insert was 10, 11, 12, or 14 mm in 120, 24, 59, and 5 knees, respectively (Table 5). Mean adjustment from the standardized surgeon-entered 10 mm starting value was 0.78 ± 1.02 mm, and the 10 mm starting value was retained in 57.7% of knees. In exploratory clustered models, no statistically significant association was observed between absolute preoperative radiographic HKA and continuous adjustment (0.056 mm per 5°, 95% CI −0.122 to 0.235; *p* = 0.538) or adjustment of at least 2 mm (OR 1.27, 95% CI 0.90 to 1.79; *p* = 0.167).

## 4. Discussion

The principal finding of this study was a systematic cross-context discrepancy between post-trial-component intraoperative ROSA HKA and postoperative standing weight-bearing HKA after RATKA. Mean bias was −1.75°, conventional LoA extended from −6.36° to 2.85°, and ICC(A,1) was 0.472. Absolute differences exceeded 2° in 45.7% and 3° in 29.8% of knees, and the negative regression slope demonstrated angle-dependent bias. The post-trial measurement was obtained after final soft-tissue balancing with the same insert thickness used for final implantation, and no subsequent component-position or insert-thickness changes were made; therefore, later intraoperative modification is unlikely to explain the discrepancy. Because no rigid positioning device or goniometer was used, small variations in ankle position or limb rotation may have contributed. The remaining difference may also reflect the transition to standing weight bearing, passive soft-tissue behavior, the distinction between a balanced trial construct and a cemented final construct, postoperative limb behavior, and radiographic acquisition. The present study cannot determine the relative contribution of each factor. One-knee-per-patient sensitivity analyses yielded similar estimates, supporting the robustness of the primary findings to within-patient dependence from bilateral procedures.

The HKA findings highlight the distinction between differently timed and differently loaded measurements. The secondary comparison showed a high absolute-agreement ICC of 0.956 between pre-cut intraoperative ROSA HKA and preoperative standing radiographic HKA; however, this coexisted with a mean bias of 0.97° and LoA from −2.45° to 4.38°. The pre-cut value was obtained after surgery began and bony registration was completed, but before meniscal removal, osteophyte removal, or bone resection, with the patient supine and the knee in full extension and without intentional axial loading or varus-valgus stress. It should not be interpreted as an independent validation of image-based preoperative planning. For the primary comparison, the regression slope of −0.569 indicates that ROSA values increasingly converged toward neutral relative to standing radiographs as the paired mean increased. Based on the fitted regression equation, a paired mean HKA of 0° corresponded to an expected ROSA-minus-radiographic difference of approximately −0.7°, whereas a paired mean of 5° varus corresponded to an expected difference of approximately −3.5°. Thus, the discrepancy was not constant but became more pronounced as the magnitude of varus alignment increased. For ROSA users, post-trial HKA should be interpreted as an intraoperative indicator of alignment trend and not as a fixed patient-level predictor of postoperative standing HKA, given the proportional bias and wide limits of agreement.

The broader concept that intraoperative robotic measurements and postoperative full-length standing radiographs are related but should not automatically be treated as interchangeable is consistent with prior work. A MAKO study also demonstrated systematic differences between intraoperative robotic measurements and postoperative full-leg radiographs, with postoperative full-leg radiographs demonstrating less residual coronal varus than intraoperative robotic measurements [17]; in the present ROSA cohort, postoperative standing radiographs tended to show greater varus relative to the intraoperative ROSA measurement as varus increased. Differences in robotic platform, imaging workflow, alignment targets, soft-tissue balancing strategy, patient position, trial-versus-final construct, and weight-bearing conditions may contribute. Coronal alignment can also differ between full-length weight-bearing radiographs and non-weight-bearing CT-based robotic planning [18]. Accordingly, post-trial ROSA HKA and postoperative standing HKA should be treated as measurements obtained under different conditions rather than as substitutes for one another.

This distinction is clinically relevant because post-trial-component intraoperative ROSA HKA is recorded under intraoperative supine conditions, whereas postoperative standing HKA reflects alignment under weight-bearing conditions. Postoperative alignment may be influenced by residual flexion, pain-avoidance stance, asymmetric loading, knee flexion, limb rotation, patellar orientation, patient-specific soft-tissue behavior, and the timing of imaging [5,12,25,26,27,28,29,30,31]. Axial limb rotation and knee flexion can alter radiographic lower-limb alignment, and robot-assisted intraoperative data show that HKA may vary throughout knee motion [30,31,32]. Robotic measurements can standardize intraoperative references, while final standing alignment remains influenced by soft-tissue balance, limb posture, knee motion, and weight-bearing conditions.

Load-dependent osseous deformation is another biomechanically plausible contributor to differences between intraoperative and standing HKA. Yang et al. reported posterior tibial bending of approximately 0.15–1.30°, medial bending of 0.38–0.90°, and external tibial torsion of 0.67–1.66° during walking in healthy participants [33]. These dynamic osseous measurements cannot be directly extrapolated to static postoperative standing HKA. More directly relevant to the present comparison, Brown et al. reported a mean 1.9° shift toward varus from supine to bipedal stance in TKA knees [34], whereas Zahn et al. reported a concomitant approximately 2° shift from valgus to varus between postoperative standing assessments associated with greater limb loading [35]. These findings support loading and positioning as plausible contributors but do not isolate osseous deformation or fully explain the present limits of agreement. Importantly, the observed global HKA discrepancy should not be interpreted as evidence of inaccurate robotic bone-cut execution or component positioning because component-to-bone alignment and achieved post-cut angles were not directly assessed. Even with accurate component placement, standing HKA may differ from intraoperative measurements because of weight-bearing and soft-tissue behavior, while knee flexion, axial rotation, and joint-line convergence can further alter measured coronal alignment [31,32,33]. Moreover, because medial and lateral ligament tensions are neither necessarily equal nor constant throughout flexion, complete compartmental symmetry was not the balancing objective [20]. Instead, gap patterns in full extension and at 90° of flexion guided component positioning and bone resection within the described alignment boundaries. Nevertheless, HKA agreement was evaluated only in extension because coronal HKA measurements during flexion were unavailable.

The component-sizing findings should be interpreted separately from bone-cut or component-position accuracy. Robotic-assisted TKA has been reported to reduce alignment outliers and improve the reproducibility of component positioning compared with conventional techniques [12,13]. Cadaveric and comparative studies have reported small angular and resection-thickness errors and fewer alignment outliers with robotic assistance [4,12,13,14,15,36], and a prospective randomized CT study also found improved component and limb alignment [37]. In the present study; however, femoral exact size agreement was 42.3%, and planned sizes were more often larger than smaller relative to the implanted component (41.8% versus 15.9%). Nevertheless, 88.5% of cases were within one size level and all were within two size levels, indicating strong ordinal association despite limited exact agreement. A plausible explanation for the tendency toward planned oversizing is that the preoperative image-based estimate may not perfectly reproduce the femoral dimensions encountered after intraoperative exposure and osteophyte removal. Differences between the preoperative image-derived condylar contour and the final intraoperative anatomy, together with intraoperative assessment of mediolateral component fit, gap balance, and component positioning, may contribute to selection of a smaller final femoral component. These mechanisms were not directly measured in the present study and should therefore be regarded as explanatory hypotheses rather than confirmed causes. Clinically, the planned femoral size should be regarded as an initial sizing reference rather than a definitive implant prescription, and final selection should continue to incorporate direct assessment of femoral anatomy, component fit, gap balance, and component positioning. Tibial exact agreement was 80.8%, with linearly weighted kappa 0.815. Because postoperative CT and direct bone-cut execution measurements were not available, these sizing results should not be interpreted as evidence of final component-position or osseous-execution accuracy.

Unlike component sizing, final polyethylene insert thickness depended on intraoperative assessment. In this workflow, 10 mm was a standardized starting value entered by the operating surgeon, not a mandatory system default or an individualized system prediction, and implanted thickness ranged from 10 to 14 mm. The 10 mm starting value was retained in 57.7% of knees. Exploratory models did not identify a statistically significant association between preoperative HKA magnitude and adjustment. Insert selection remains plausibly influenced by soft-tissue tension, flexion-extension gap balance, and intraoperative stability assessment [38,39,40,41,42,43,44]. Planned-to-measured differences may persist despite accurate bone preparation, and balancing may leave residual laxity patterns outside full extension and 90° of flexion [16,26]. Adjustment should therefore be interpreted relative to the standardized surgeon-entered starting value and as a reflection of individualized intraoperative balancing, not as system prediction error.

Although a systematic and angle-dependent difference between post-trial ROSA HKA and postoperative standing radiographic HKA was demonstrated, the clinical significance of this discrepancy remains uncertain. The present study did not evaluate patient-reported outcomes, functional outcomes, complications, or implant survivorship, and no clinically acceptable threshold for the HKA difference or limits of agreement was prespecified. Accordingly, these findings characterize measurement disagreement between the two conditions but do not demonstrate that the observed discrepancy is associated with inferior clinical outcomes or that either measurement modality is clinically superior.

Several methodological features support the robustness of these findings, including consecutive enrollment, consistent operative and radiographic workflows, and the use of Bland–Altman analysis and absolute-agreement ICC rather than correlation alone. Within-patient dependence from bilateral procedures was addressed through patient-clustered inference and one-knee-per-patient sensitivity analyses. Moreover, although distributional assessment identified modest departures from normality, empirical percentile limits were similar to the conventional Bland–Altman limits, indicating that these departures did not materially affect the interpretation of agreement.

Several limitations warrant consideration. First, this retrospective single-center study involved one surgeon, one robotic platform, and one implant system; the predominantly varus cohort and pooling of posterior-stabilized and medial-congruent constructs further limit generalizability and subgroup inference. Although bilateral knees were addressed using patient-clustered analyses and one-knee-per-patient sensitivity analyses, the sample remained modest for subgroup comparisons. Second, only one ROSA HKA value was recorded at each intraoperative stage, precluding formal repeatability assessment. During post-trial acquisition, the ankle was positioned clinically at approximately 90° without rigid fixation or goniometric confirmation, and repeated acquisitions after repositioning were unavailable. Quantitative post-cut angular validation was not performed; Vernier caliper assessment checked resected bone thickness but not achieved cut-plane orientation. Consequently, positioning, single-measurement, and residual cut-execution variability cannot be separated from true cross-context differences. Third, the primary measurement represented the final trial rather than the cemented construct, and post-cementation HKA was unavailable. Cementation-related changes in compartment loading, component seating, or cement mantle could have contributed to the discrepancy [45], but post-cementation HKA and cement-mantle measurements were unavailable. Fourth, component alignment and rotation were not assessed using postoperative CT or component-specific radiographs. Based on the operative team’s recollection, the radiographic observer had assisted in approximately the first 15–20 ROSA procedures, although case-level participation records were unavailable and the specific knees could not be identified retrospectively. Measurements were performed using deidentified, study-coded images without access to the code linkage, clinical information, or ROSA values. These safeguards reduced, but did not eliminate, the possibility of case recognition, and an exclusion sensitivity analysis was not feasible. Repeated radiographic measurements were unavailable, precluding cohort-specific intraobserver and interobserver reliability assessment. Finally, clinical outcomes, survivorship, longitudinal alignment, prespecified clinical agreement thresholds, and a priori sample-size calculation were unavailable. Future studies should incorporate standardized limb positioning, prespecified loading conditions, repeated measurements, quantitative post-cut and post-cementation assessment, component-specific imaging, and clinical outcomes.

## 5. Conclusions

In this single-center, single-surgeon ROSA-assisted TKA cohort, post-trial intraoperative HKA and postoperative standing radiographic HKA were associated but not interchangeable, with individual cross-context differences extending over several degrees. The systematic and angle-dependent relationship precludes application of a simple patient-level correction. The magnitude of disagreement is relevant to commonly used alignment thresholds and highlights the need for future studies to standardize and report patient position, loading condition, measurement timing, post-cut validation, and measurement repeatability. However, because clinical outcomes and a prespecified acceptable-difference threshold were unavailable, these cross-context differences should not be interpreted as established patient-level clinical errors. The findings should be interpreted within the specific ROSA workflow studied and should not be assumed to generalize directly to other robotic platforms or surgical settings.

## Figures and Tables

**Figure 1:**
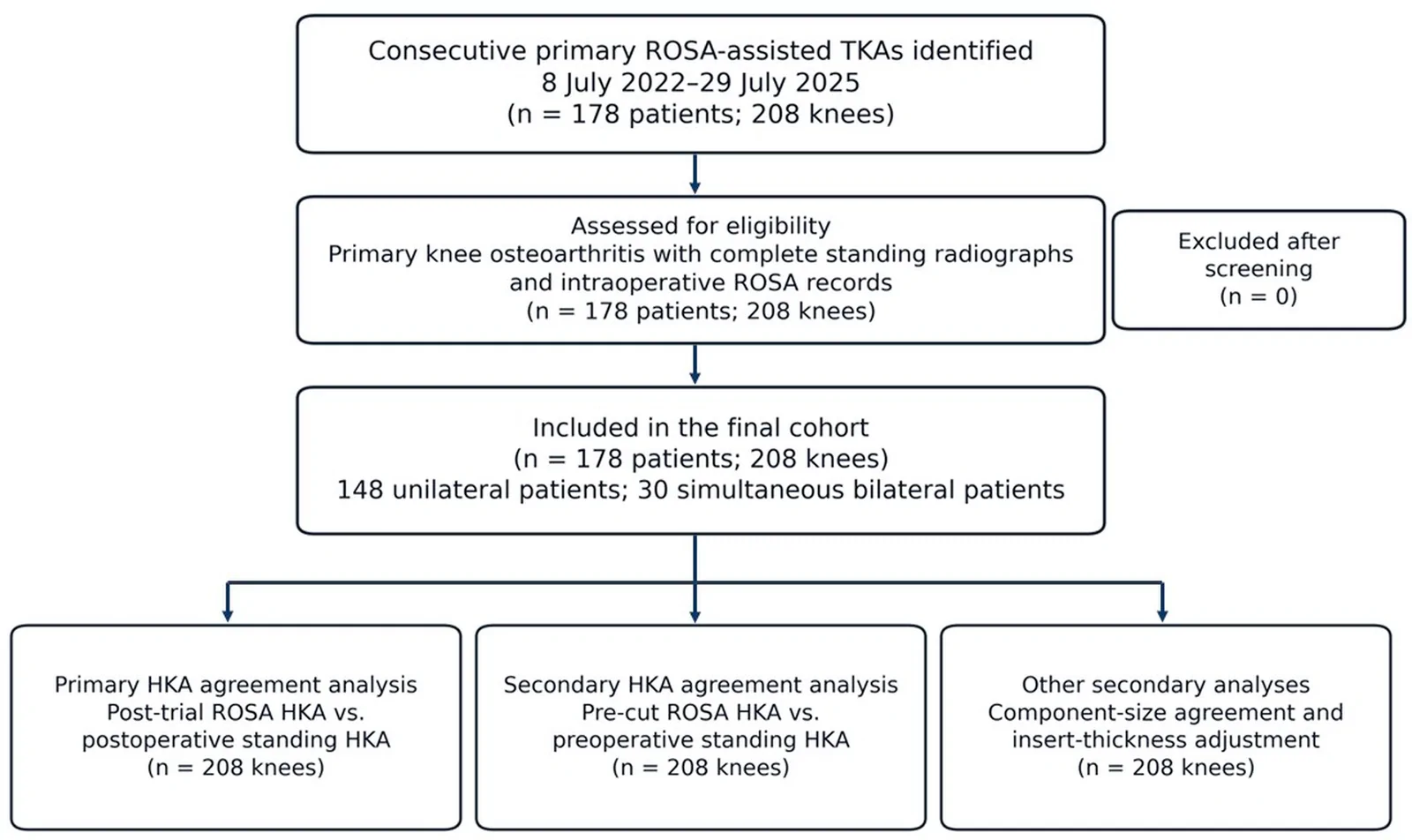
Participant flow and study analyses. Flow diagram showing the identification, eligibility assessment, inclusion, and analysis of consecutive primary ROSA-assisted total knee arthroplasties performed between 8 July 2022 and 29 July 2025. All 178 eligible patients (208 knees), including 148 unilateral and 30 simultaneous bilateral cases, were included in the primary and secondary analyses; no cases were excluded after screening.

**Figure 2:**
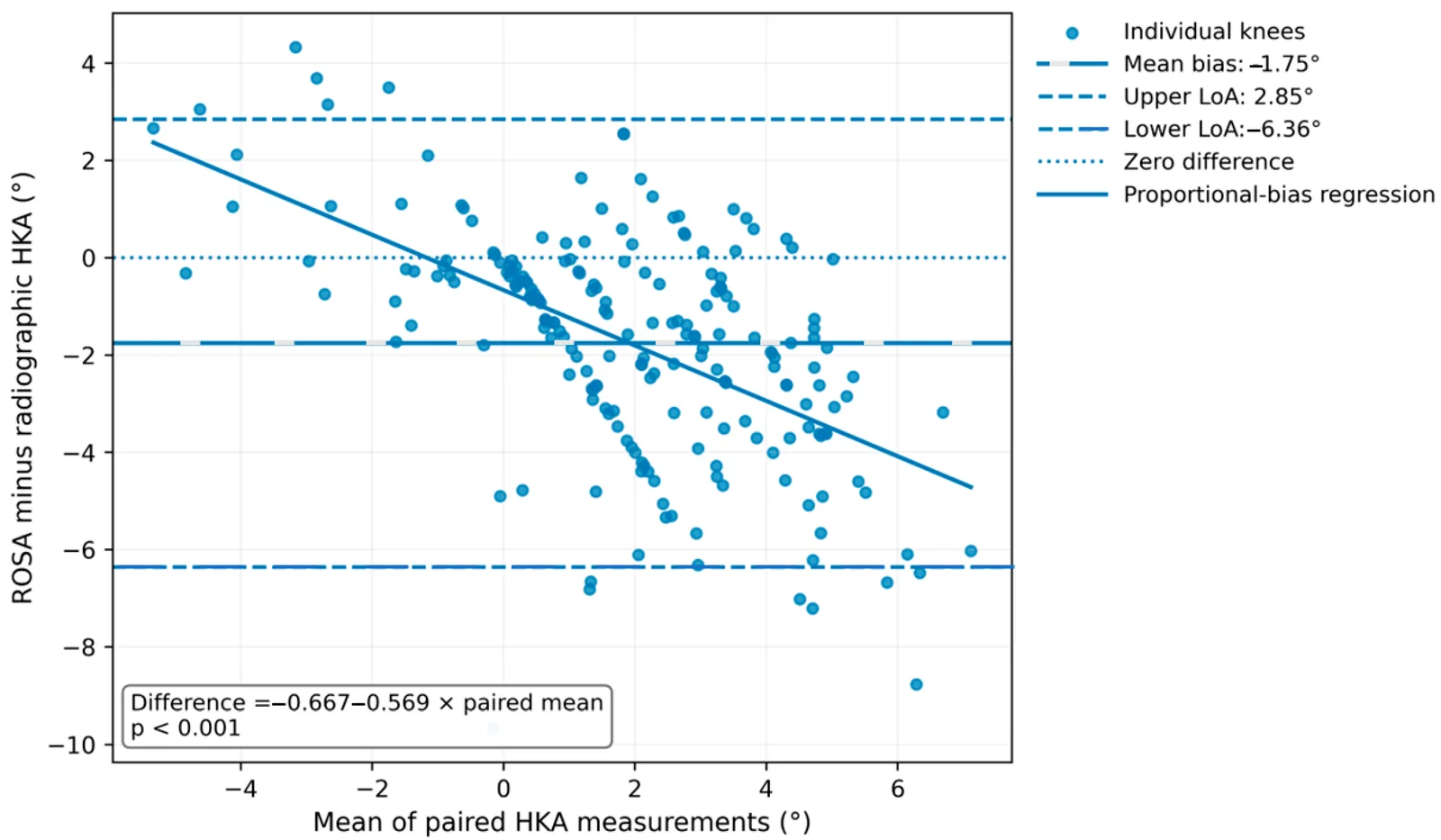
Bland–Altman plot for post-trial-component intraoperative ROSA HKA versus postoperative standing radiographic HKA with proportional-bias regression. Difference is defined as ROSA minus radiographic HKA. Points above and below the zero-difference line represent intraoperative ROSA measurements that were higher and lower, respectively, than the corresponding postoperative radiographic measurements. Although differences occurred in both directions, the mean difference was negative and proportional bias was present. The solid horizontal line indicates mean bias, the dashed horizontal lines indicate the conventional 95% limits of agreement, the dotted horizontal line indicates zero difference, and the oblique line indicates the fitted proportional-bias regression. The legend is positioned outside the upper-right margin of the plotting area.

**Figure 3:**
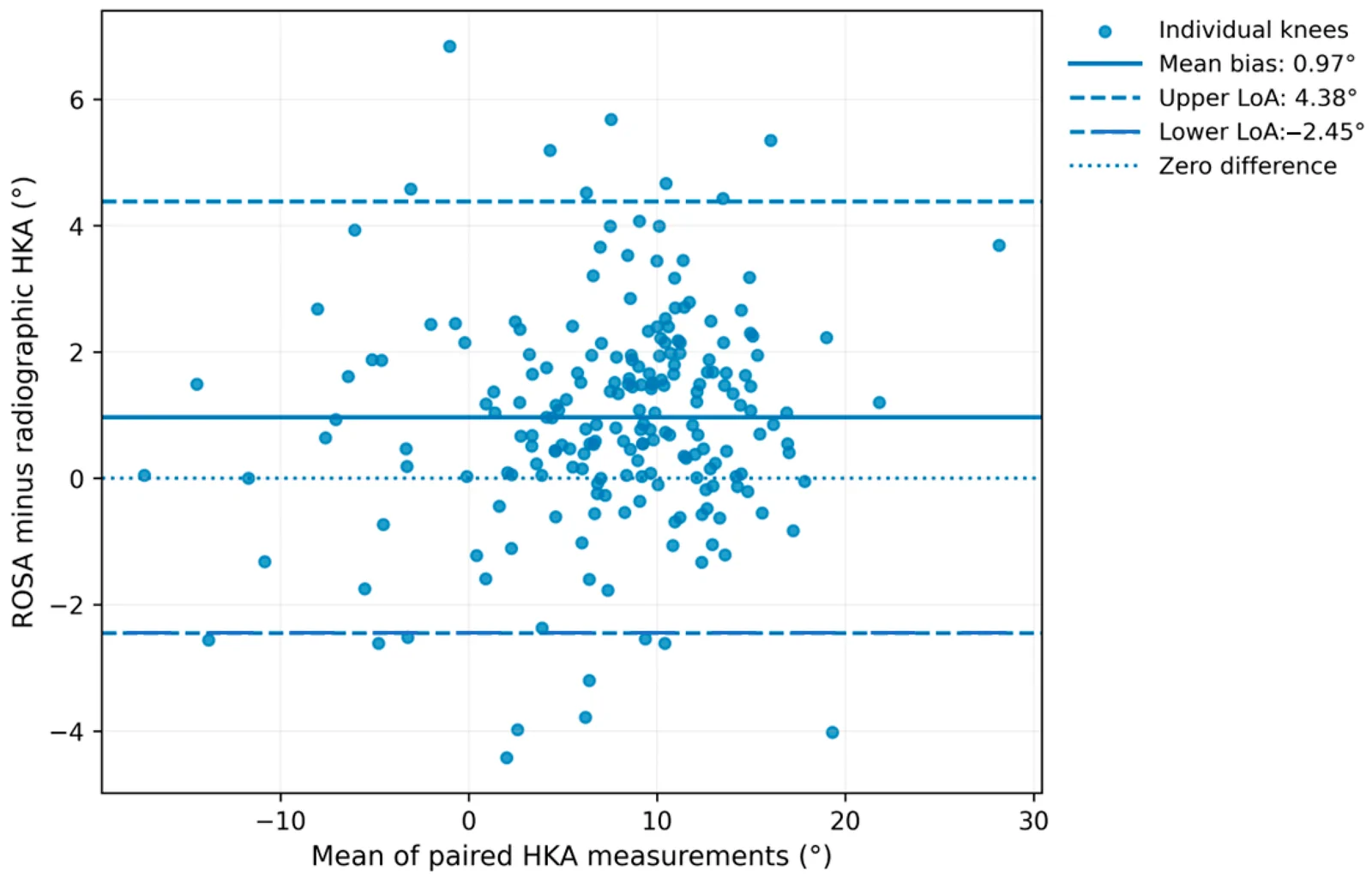
Conventional Bland–Altman plot. Bland–Altman plot for pre-cut intraoperative ROSA HKA versus preoperative standing radiographic HKA. Difference is defined as ROSA minus radiographic HKA. The solid horizontal line indicates mean bias, the dashed horizontal lines indicate the conventional 95% limits of agreement, and the dotted horizontal line indicates zero difference. The legend is positioned outside the upper-right margin of the plotting area.

**Table 1:** Patient- and knee-level characteristics.

Characteristic	Value	Unit/Denominator
Patient-level characteristics		
Patients	178	one record per patient
Age	72.4 ± 6.5	years
Female sex	132 (74.2%)	*n* = 178
Male sex	46 (25.8%)	*n* = 178
Height	155.9 ± 8.0	cm
Weight	66.0 ± 11.7	kg
Body mass index	27.1 ± 3.8	kg/m^2^
Knee-level characteristics		
Knees	208	all knees
Left side	107 (51.4%)	*n* = 208
Right side	101 (48.6%)	*n* = 208
Posterior-stabilized construct	140 (67.3%)	*n* = 208
Medial-congruent construct	68 (32.7%)	*n* = 208
Preoperative and pre-cut alignment		
Pre-cut intraoperative ROSA HKA	8.16 ± 6.81	degrees
Preoperative standing radiographic HKA	7.19 ± 6.61	degrees
Preoperative varus/neutral/valgus	168/20/20	exploratory ±3° categories
Trial and postoperative alignment		
Post-trial-component intraoperative ROSA HKA	1.03 ± 1.90	degrees
Postoperative standing radiographic HKA	2.79 ± 3.09	degrees
Postoperative varus/neutral/valgus	104/94/10	exploratory ±3° categories

Values are mean ± standard deviation or *n* (%). HKA, hip-knee-ankle angle. Exploratory alignment categories were defined as valgus < −3°, neutral −3° to +3°, and varus > +3°.

**Table 2:** Agreement between intraoperative ROSA and standing radiographic HKA measurements.

Measure	Primary Comparison	Secondary Comparison
Mean bias, °	−1.75 (−2.08 to −1.43)	0.97 (0.72 to 1.22)
Lower 95% LoA, °	−6.36 (−7.00 to −5.70)	−2.45 (−2.92 to −1.95)
Upper 95% LoA, °	2.85 (2.34 to 3.35)	4.38 (3.89 to 4.87)
ICC(A,1)	0.472 (0.379 to 0.557)	0.956 (0.937 to 0.969)
Spearman ρ	0.626 (0.520 to 0.715)	0.954 (0.931 to 0.968)
Mean absolute difference, °	2.23 (1.97 to 2.49)	1.54 (1.37 to 1.73)
Within 2°	54.3% (47.2% to 61.3%)	72.6% (66.4% to 78.5%)
Within 3°	70.2% (63.5% to 76.5%)	88.0% (83.3% to 92.3%)
Proportional-bias slope	−0.569 (−0.683 to −0.456); *p* < 0.001	0.031 (−0.009 to 0.071); *p* = 0.124

°, degree. Values are estimates with 95% confidence intervals in parentheses. The primary comparison was post-trial-component intraoperative ROSA HKA versus postoperative standing radiographic HKA; the secondary comparison was pre-cut intraoperative ROSA HKA versus preoperative standing radiographic HKA. Difference was defined as ROSA minus radiographic HKA. LoA, limits of agreement. Confidence intervals used patient-cluster bootstrap resampling, except for regression coefficients, which used patient-cluster-robust standard errors.

**Table 3:** Femoral component-size agreement.

Metric	Estimate (95% CI)
Exact agreement	42.3% (35.5% to 49.3%)
Planned size smaller than implanted	15.9% (11.0% to 21.1%)
Planned size larger than implanted	41.8% (34.8% to 49.0%)
Mean absolute size-level difference	0.69 (0.60 to 0.79)
Within 1 size level	88.5% (83.7% to 92.8%)
Within 2 size levels	100.0% (100.0% to 100.0%)
Linearly weighted kappa	0.631 (0.570 to 0.685)
Spearman ρ	0.851 (0.802 to 0.888)

CI, confidence interval. Confidence intervals were obtained by patient-cluster bootstrap resampling. Planned size smaller/larger refers to the planned size relative to the implanted size. Kappa describes agreement; Spearman ρ describes ordinal association.

**Table 4:** Tibial component-size agreement.

Metric	Estimate (95% CI)
Exact agreement	80.8% (75.0% to 86.3%)
Planned size smaller than implanted	6.7% (3.4% to 10.2%)
Planned size larger than implanted	12.5% (7.8% to 17.5%)
Mean absolute size-level difference	0.21 (0.15 to 0.28)
Within 1 size level	98.6% (96.7% to 100.0%)
Within 2 size levels	99.5% (98.5% to 100.0%)
Linearly weighted kappa	0.815 (0.760 to 0.865)
Spearman ρ	0.908 (0.863 to 0.945)

CI, confidence interval. Confidence intervals were obtained by patient-cluster bootstrap resampling. Planned size smaller/larger refers to the planned size relative to the implanted size. Kappa describes agreement; Spearman ρ describes ordinal association.

**Table 5:** Implanted insert-thickness distribution and adjustment from the standardized surgeon-entered 10 mm starting value.

Measure	Result
10 mm	120 (57.7%)
11 mm	24 (11.5%)
12 mm	59 (28.4%)
14 mm	5 (2.4%)
Adjustment from surgeon-entered 10 mm starting value	
Mean adjustment, mm	0.78 (95% CI, 0.64 to 0.92)
10 mm starting value retained	57.7% (95% CI, 51.0% to 64.4%)

Values are *n* (%) unless otherwise specified. CI, confidence interval. The 10 mm value was a standardized starting value entered by the operating surgeon rather than a mandatory system default or a system-generated patient-specific prediction.

## Data Availability

The data supporting the findings of this study are not publicly available because of patient privacy and institutional restrictions. Data may be available from the corresponding author upon reasonable request and subject to approval by the Institutional Review Board and institutional requirements. The analysis code may be obtained from the corresponding author upon reasonable request.

## Supplementary Materials

The following supporting information can be downloaded at: https://www.mdpi.com/article/10.3390/jcm15197432/s1, Table S1: Complete HKA agreement estimates; Table S2: Exploratory ±3° HKA-category confusion matrices; Table S3: Femoral planned-by-implanted component-size matrix; Table S4: Tibial planned-by-implanted component-size matrix; Table S5: Bowker patient-cluster permutation tests; Table S6: One-knee-per-patient sensitivity analyses; Table S7: Regression-based Bland–Altman estimates; Table S8: Exploratory insert-adjustment models; Table S9: Distributional diagnostics and sensitivity analyses for Bland–Altman differences.
