# Supplementary material for: Agreement and Systematic Differences Between Intraoperative ROSA Hip-Knee-Ankle Measurements and Standing Radiographic Alignment After Robotic-Assisted Total Knee Arthroplasty: A Retrospective Agreement Study
Source: J Clin Med. 2026 Sep 24;15(19):7432. doi: 10.3390/jcm15197432 (PMC13666193; doi:10.3390/jcm15197432)
Supplement: Supplementary file 1 [file jcm-15-07432-s001.zip › jcm-4477092-supplementary.pdf]

JCM Supplementary Materials

Supplementary Materials

Agreement and Systematic Differences Between Intraoperative ROSA Hip-Knee-Ankle Measurements and Standing Radiographic Alignment After Robotic-Assisted Total Knee Arthroplasty: A Retrospective Agreement Study

Difference is defined as ROSA minus radiographic HKA. Confidence intervals use patient-cluster bootstrap unless otherwise specified.

Table S1. Complete HKA agreement estimates.

Comparison	Metric	Estimate	95% CI
Primary	Mean bias, degrees	-1.75	-2.08 to -1.43
Primary	SD of differences, degrees	2.35	2.09 to 2.60
Primary	Conventional lower LoA, degrees	-6.36	-7.00 to -5.70
Primary	Conventional upper LoA, degrees	2.85	2.34 to 3.35
Primary	ICC(A,1)	0.472	0.379 to 0.557
Primary	Spearman rho	0.626	0.520 to 0.715
Primary	Mean absolute error, degrees	2.23	1.97 to 2.49
Primary	Median absolute error, degrees	1.65	1.38 to 2.10
Primary	Within 1 degree	34.1%	27.5% to 41.1%
Primary	Within 2 degrees	54.3%	47.2% to 61.3%
Primary	Within 3 degrees	70.2%	63.5% to 76.5%
Primary	Exploratory 3-category weighted kappa	0.263	0.163 to 0.367
Secondary	Mean bias, degrees	0.97	0.72 to 1.22
Secondary	SD of differences, degrees	1.74	1.52 to 1.95
Secondary	Conventional lower LoA, degrees	-2.45	-2.92 to -1.95
Secondary	Conventional upper LoA, degrees	4.38	3.89 to 4.87
Secondary	ICC(A,1)	0.956	0.937 to 0.969
Secondary	Spearman rho	0.954	0.931 to 0.968
Secondary	Mean absolute error, degrees	1.54	1.37 to 1.73
Secondary	Median absolute error, degrees	1.34	1.08 to 1.51
Secondary	Within 1 degree	39.4%	32.8% to 46.3%
Secondary	Within 2 degrees	72.6%	66.4% to 78.5%
Secondary	Within 3 degrees	88.0%	83.3% to 92.3%
Secondary	Exploratory 3-category weighted kappa	0.855	0.762 to 0.926

Table S2. Exploratory ±3° HKA-category confusion matrices.

Comparison	ROSA row	Radiographic valgus	Radiographic neutral	Radiographic varus
Primary	Valgus	4	1	1
Primary	Neutral	6	88	74
Primary	Varus	0	5	29
Secondary	Valgus	17	1	0
Secondary	Neutral	3	12	3
Secondary	Varus	0	7	165

Valgus < -3°; neutral -3° to +3°; varus > +3°. Rows are ROSA categories and columns are radiographic categories.

Table S3. Femoral planned-by-implanted component-size matrix.

Planned / implanted	3	4	5	6	7	8	9	10	11	12
3	1	0	0	0	0	0	0	0	0	0
4	4	5	4	1	0	0	0	0	0	0

Planned / implanted	3	4	5	6	7	8	9	10	11	12
5	2	10	20	6	0	0	0	0	0	0
6	0	3	12	17	11	1	0	0	0	0
7	0	0	4	21	24	5	0	0	0	0
8	0	0	0	2	9	11	4	0	0	0
9	0	0	0	0	4	6	4	1	0	0
10	0	0	0	0	0	4	3	3	0	0
11	0	0	0	0	0	0	2	0	3	0
12	0	0	0	0	0	0	0	1	0	0

Bowker patient-cluster permutation p = 0.003; 10,000 permutations. For each permutation, planned and implanted labels were swapped together within a sampled patient cluster according to one Bernoulli decision, preserving dependence between bilateral knees.

Table S4. Tibial planned-by-implanted component-size matrix.

Planned / implanted	C	D	E	F	G	H
C	92	9	1	0	0	0
D	4	45	3	1	0	0
E	0	4	23	0	0	0
F	0	0	9	5	0	0
G	0	1	0	7	3	0
H	0	0	0	0	1	0

Bowker patient-cluster permutation p < 0.001; 10,000 permutations. For each permutation, planned and implanted labels were swapped together within a sampled patient cluster according to one Bernoulli decision, preserving dependence between bilateral knees.

Table S5. Bowker patient-cluster permutation tests.

Component	Bowker statistic	Degrees of freedom	Cluster permutation p
Femoral	32.573	15	p = 0.003
Tibial	22.066	8	p < 0.001

Table S6. One-knee-per-patient sensitivity analyses.

Comparison	Metric	Fixed selection	Repeated median	Stability interval
Primary	bias	-1.774	-1.790	-1.850 to -1.727
Primary	sd difference	2.442	2.431	2.401 to 2.460
Primary	loa lower	-6.559	-6.555	-6.645 to -6.462
Primary	loa upper	3.012	2.975	2.892 to 3.054
Primary	icc a1	0.467	0.467	0.456 to 0.477
Primary	spearman rho	0.614	0.618	0.596 to 0.638
Secondary	bias	0.954	0.943	0.888 to 0.993
Secondary	sd difference	1.760	1.764	1.722 to 1.808
Secondary	loa lower	-2.494	-2.518	-2.599 to -2.436
Secondary	loa upper	4.403	4.400	4.295 to 4.513
Secondary	icc a1	0.958	0.957	0.955 to 0.959
Secondary	spearman rho	0.958	0.955	0.951 to 0.959

Stability intervals summarize 1,000 random one-knee selections and are not sampling confidence intervals.

Table S7. Regression-based Bland-Altman estimates.

Comparison	Parameter	Estimate	95% CI	p-value
Primary	Intercept	-0.667	-1.004 to -0.330	p < 0.001
Primary	Slope	-0.569	-0.683 to -0.456	p < 0.001
Primary	10th percentile (paired mean - 0.88°) expected difference	-0.16	-0.57 to 0.22	
Primary	10th percentile (paired mean - 0.88°) conditional lower LoA	-3.26	-4.28 to -2.35	
Primary	10th percentile (paired mean -	2.93	2.23 to 3.64	

Comparison	Parameter	Estimate	95% CI	p-value
	0.88°) conditional upper LoA			
Primary	50th percentile (paired mean 2.03°) expected difference	-1.82	-2.10 to -1.56	
Primary	50th percentile (paired mean 2.03°) conditional lower LoA	-5.44	-6.02 to -4.82	
Primary	50th percentile (paired mean 2.03°) conditional upper LoA	1.79	1.33 to 2.19	
Primary	90th percentile (paired mean 4.73°) expected difference	-3.36	-3.80 to -2.91	
Primary	90th percentile (paired mean 4.73°) conditional lower LoA	-7.45	-8.24 to -6.58	
Primary	90th percentile (paired mean 4.73°) conditional upper LoA	0.73	0.04 to 1.37	
Secondary	Intercept	0.730	0.310 to 1.150	p < 0.001
Secondary	Slope	0.031	-0.009 to 0.071	p = 0.124
Secondary	10th percentile (paired mean -0.81°) expected difference	0.70	0.28 to 1.17	
Secondary	10th percentile (paired mean -0.81°) conditional lower LoA	-2.69	-3.23 to -2.05	
Secondary	10th percentile (paired mean -0.81°) conditional upper LoA	4.10	3.44 to 4.73	
Secondary	50th percentile (paired mean 9.04°) expected difference	1.01	0.76 to 1.26	
Secondary	50th percentile (paired mean 9.04°) conditional lower LoA	-2.39	-2.85 to -1.87	
Secondary	50th percentile (paired mean 9.04°) conditional upper LoA	4.41	3.90 to 4.88	
Secondary	90th percentile (paired mean 14.53°) expected difference	1.18	0.83 to 1.51	
Secondary	90th percentile (paired mean 14.53°) conditional lower LoA	-2.22	-2.77 to -1.63	
Secondary	90th percentile (paired mean 14.53°) conditional upper LoA	4.58	4.03 to 5.09	

Primary conditional LoA use a varying-width absolute-residual model; secondary conditional LoA use approximately constant residual variance.

Table S8. Exploratory insert-adjustment models.

Outcome	Effect per 5° absolute preoperative HKA	95% CI	p-value
Continuous adjustment, mm	0.056	-0.122 to 0.235	p = 0.538
Adjustment ≥2 mm, odds ratio	1.27	0.90 to 1.79	p = 0.167

Both models account for patient clustering. No statistically significant association was observed.

Table S9. Distributional diagnostics and sensitivity analyses for Bland-Altman differences.

Comparison	Skewness	Excess kurtosis	Shapiro-Wilk W	p-value	Conventional 95% LoA, degrees	Empirical 2.5th-97.5th percentile, degrees
Primary	-0.423	0.477	0.982	0.010	-6.36 to 2.85	-6.68 to 2.64
Secondary	-0.084	1.302	0.977	0.002	-2.45 to 4.38	-2.61 to 4.57

Primary comparison: post-trial-component intraoperative ROSA HKA versus postoperative standing radiographic HKA. Secondary comparison: pre-cut intraoperative ROSA HKA versus preoperative standing radiographic HKA. Difference was defined as ROSA minus radiographic HKA. Distributional assessment included histogram and normal Q-Q plot inspection, skewness, excess kurtosis, and the Shapiro-Wilk test. Because formal normality tests may detect minor departures from normality in moderate-to-large samples, graphical distribution and tail behavior were considered together with the formal test. Empirical percentile limits are shown as a sensitivity analysis and did not materially alter the interpretation of the conventional Bland-Altman limits of agreement.
